# The effect of bean origin and temperature on grinding roasted coffee

**DOI:** 10.1038/srep24483

**Published:** 2016-04-18

**Authors:** Erol Uman, Maxwell Colonna-Dashwood, Lesley Colonna-Dashwood, Matthew Perger, Christian Klatt, Stephen Leighton, Brian Miller, Keith T. Butler, Brent C. Melot, Rory W. Speirs, Christopher H. Hendon

**Affiliations:** 1 Meritics Ltd., 1 Kensworth Gate, Dunstable, LU6 3HS, United Kingdom; 2 Colonna and Smalls, 6 Chapel Row, Bath, BA1 1HN, United Kingdom; 3 St Ali/Sensory Lab, 12-18 Yarra Pl, South Melbourne, Victoria, 3205, Australia; 4 Mahlkönig GmbH & Co.KG, Tilsiter Str. 142, 22047 Hamburg, Germany; 5 Has Bean Coffee Ltd., Unit 16, Ladford Covert, Stafford, ST18 9QL, United Kingdom; 6 Department of Chemistry, University of Bath, BA2 7AY, Bath, United Kingdom; 7 Department of Chemistry, University of Southern California, Los Angeles, CA 90089, United States of America; 8 School of Physics, The University of Melbourne, Victoria, 3010, Australia; 9 Department of Chemistry, Massachusetts Institute of Technology, Cambridge, MA 02139, United States of America

## Abstract

Coffee is prepared by the extraction of a complex array of organic molecules from the roasted bean, which has been ground into fine particulates. The extraction depends on temperature, water chemistry and also the accessible surface area of the coffee. Here we investigate whether variations in the production processes of single origin coffee beans affects the particle size distribution upon grinding. We find that the particle size distribution is independent of the bean origin and processing method. Furthermore, we elucidate the influence of bean temperature on particle size distribution, concluding that grinding cold results in a narrower particle size distribution, and reduced mean particle size. We anticipate these results will influence the production of coffee industrially, as well as contribute to how we store and use coffee daily.

Second only to oil, coffee is the most valuable legally traded commodity. There are two biologically dissimilar species of coffee grown for consumption; *Coffea canephora* (robusta) and *Coffea arabica* (arabica)^1^. Whilst robusta is both less chemically complex and less flavoursome than arabica, it benefits from being feasibly grown at low altitude and is pest resistant. However, over 60% of the global coffee consumption is of arabica. In 2014, Brazil and Colombia combined to produce over 3.5 million tonnes of green arabica^2^, with Ethiopia and other African and Central American producers also making significant contributions. Including countries like Vietnam which almost exclusively produces robusta, global coffee production amounts to 8.5 million tonnes annually.

With the exception of unusual green coffee medicinal and dietary preparations, coffee is not typically consumed as a solid but rather an extract from the roasted seed^3^,^4^,^5^,^6^,^7^,^8^,^9^. Coffee beans are imported, roasted, ground and then brewed (including instant coffee) in coffee shops and homes. In such a valuable industry, the quality and yield of the product is paramount. However, there are many variables that influence the flavour, yield and overall enjoyment of this mass consumed beverage^10^. The challenges associated with ensuring coffee quality can be divided into two categories i) variables associated with the country of origin and ii) variables associated with consumption.

Besides typical botanical influences including climate and altitude, there are two general considerations that affect the coffee at the origin: the variety of coffee (*e.g*. Typica, Pacamara, Geisha)^11^ and the processing method (*i.e*. washed, pulped and natural). The variety defines chemical characteristics of the bean, and also the conditions in which it may be grown. Ideally, the fruit of the coffee bean should not ripen more rapidly than the ovum develops, otherwise the seed is lacking chemical complexity. Conversely, the fruit should be able to ripen in variable climate conditions thereby permitting the formation of the seed. Genetic variety hybrids are now ubiquitous and often feature the best of both of the parent varieties^12^,^13^.

Irrespective of the variety, all coffee is processed in one of three general methods. The washing (or wet) process is the most common, and uses water to remove the skin and fruit of the cherry, leaving only the seeds to dry in the sun. The pulped (pulped natural) processing method removes the skin from the cherry, but does not fully remove the mucilage. This then forms a sun-hardened sugar-rich shell around the parchment (the thin protective layer for the seed). The natural process is simply the sun-drying of the coffee cherries with both seed and fruit intact.

Whilst the processing method used has a profound impact on flavour, the chemical mechanisms which dictate these differences are not well-understood. Regardless of the cherry processing method, after drying the beans are hulled, which exposes the bean by removing all the dry parchment, mucilage, or skin. The green coffee beans are then transported to roasteries, where the roaster develops a roast profile with the aim of producing the most flavoursome cup to their palate. The roast profile is a two variable problem of temperature and time, but due to limitations of roasting equipment and the inhomogeneity of heat transfer into green coffee^14^, the development of a roast profile is more artistic than scientific, although there is certainly room for improvement in this area.

The roast profile presented in Fig. 1 shows the measured roaster temperature as the roasting progresses for the particular Tanzanian coffee listed in Table 1. The chemical constituents of roasted coffee depend on the temperatures of green coffee molecular decomposition. The generation and concentration control of these compounds is achieved through fine tuning of the roast profile^15^,^16^,^17^. Whilst most compounds in roasted coffee are likely Maillard products (an example of which is not shown in Fig. 1)^18^, we present various pathways that permit the formation of acids, phenolic compounds, and also the cleavage of cellulose into sugar-related products like levoglucosan. The left-most process in Fig. 1 shows an example of decomposition of a chlorogenic acid (a group of molecules contributing to 66% of the acidity in green coffee) through low temperature hydrolysis, in which the formation of products depend on the water content within the seed^19^,^20^.

Undoubtedly the extent and quality of extraction is dictated by the accessibility of the organic molecules contained within roasted coffee. Many factors influence the total amount, and relative proportions of the different organic molecules extracted, including temperature of brew, water chemistry and water-to-coffee ratio^21^,^22^,^23^,^24^. Here, however, we are specifically concerned with physical method of increasing accessible surface area; *i.e*. the effect of the grinder.

Whilst routine in the pharmaceutical industry, it is challenging to both design and execute a grind to a homogeneous particle size in a coffee shop. This, however, is of critical importance in coffee brewing because variable accessible surface area causes the small particles to extract more rapidly relative to larger ones. As a result, brewing coffee is challenging with variable particle size, especially in espresso-style pressurised brews, where packing effects become important^25^,^26^. Given the importance of particle size, we assess if bean origin, cherry processing method, and roast profile have any significant effect on the particle size distribution of the ground coffee.

Additionally, it was suspected that the temperature of the beans could also influence the bean fracturing dynamics, and therefore the final size distribution. Whilst ideally the beans and burrs would both be brought to the desired temperature, controlled active heating or cooling of the burrs is not presently feasible. To investigate the temperature effects we pursued the controlled cooling of the coffee itself. Given that many people store coffee in the refrigerator or freezer (if devoid of water vapour this is a chemically reasonable method of storage), we examine if varying bean temperature results in an observable modulation of grind distribution.

## Methods

For this study, it was assumed that the most important property of the ground coffee which can vary in the grinding process is the distribution of particle sizes. Whilst it is possible that particle shape may have an effect on the final extracted brew, it is difficult to see how this can be reliably controlled on the micrometer scale, and it is likely that most ground coffee has a similar spread of particle shapes.

The first set of experiments explored if the origin, type, or processing method of the bean had any effect on the particle size distribution, when ground under identical conditions. The second set of measurements explored if bean temperature at the time of grinding had any effect on produced particle size distribution.

To probe these effects we employed laser diffraction particle size analysis of roasted coffees ground on a Mahlkönig EK 43 coffee grinder.

### Laser diffraction particle size analysis

The laser diffraction particle size analysis was performed on the multiple wavelength Beckman Coulter LS13 320 MW. The instrument has a built in dark field reticule which is used to ensure correct optical alignment. An alignment check was carried out prior to every run to ensure the optimum accuracy of the particle size distribution. Particular care was taken to ensure correct optical alignment because ground coffee contains particles sizes spanning 3 orders of magnitude, including components larger than 100 *μ*m, which can be challenging to measure with diffraction based techniques since they rely on distance measurements in reciprocal space.

### Grinding

**Figure 2:**
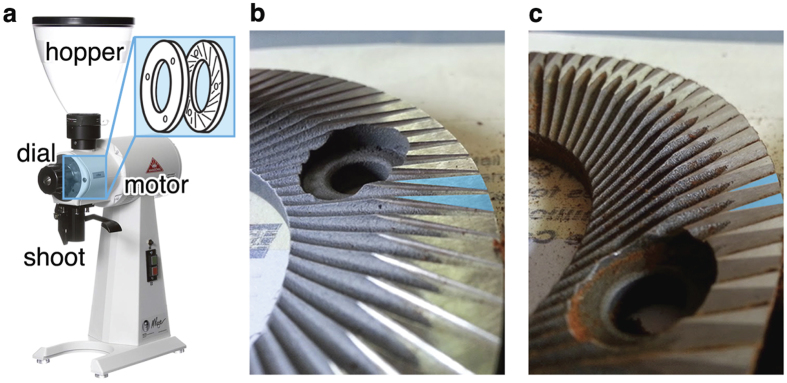
The EK 43 grinder, (**a**) consists of two burrs; one stationary and one mobile. The hopper-to-shoot path is linear resulting in minimal retention of ground coffee in the burrs and shoot. There are two types of burrs: Turkish, (**b**) and Coffee, (**c**). The primary differences between the two burrs are emphasised in blue in (**b**,**c**), respectively. The flat triangular ends are intended to polish the particulates. For this study we employed the Turkish burr set. Photographs taken by Spencer Webb.

The Mahlkönig EK 43 grinder, shown in Fig. 2, was selected for this study because it is designed to have minimum retention time between placing the coffee in the hopper and subsequent grinding. Like all grinders, the EK 43 burrs are replaceable and are susceptible to becoming misaligned (where the two grinding discs are not perfectly parallel). We had access to three separate EK 43 s on the day of testing; two which were fitted with so-called coffee burrs and one with Turkish burrs (Fig. 2). Burr alignment can initially be assessed audibly by closing the burr aperture with the grinder turned on, causing them to ‘chirp’. The pitch of the chirp provides insight into the alignment, with deeper chirps indicating more contact between the burrs and therefore better alignment. Assessing the smoothness and spread of ground particle distribution can also give information on burr alignment, though it is difficult and slow to reliably adjust alignment based on this information. We have provided one example of particle size distributions from a burr misalignment in Figure S1. Ultimately, we elected to use the grinder that was producing subjectively marketable espresso shots that day, as determined by a resident qualified Q-grader and shop owner (Maxwell Colonna-Dashwood)^27^. Experiments herein were performed with the Mahlkönig EK 43 grinder spinning at 1480 rpm and grinding with Turkish burrs.

### Coffee origin and processing

To determine if bean origin has an effect on particle size distribution after grinding, beans were tested from four countries: Guatemala, El Salvador, Tanzania, and Ethiopia. The beans had been roasted by roasteries listed in Table 1, between seven and sixteen days prior to the grinding test, and so had sufficient time for CO_2_ degassing, but were still considered ‘freshly roasted’. Further details of the four coffees considered in this study are presented in Table 1. All beans were allowed to equilibriate to room temperature (at the time, 20 °C and 79% relative humidity), densities of the roasted coffee beans were not measured. The grinder burr aperture was kept constant for all coffees throughout the experiment, fixed at 2.7 (arbitrary units) on the stock EK 43 dial. For each measurement, 20 grams of coffee was ground, and the grinder was allowed to cool for 10 minutes after each grind (returning to room temperature).

### Coffee temperature

For temperature studies, we selected the Guatemalan coffee because this particular Guatemalan crop is representative of contemporary speciality grade coffee (*i.e*. it has a favourable balance of acidity, floral complexity and overall taste). The four temperatures were achieved using the following method: 20 g of whole roasted coffee beans were placed into a paper cup, covered, and placed into either liquid nitrogen, a tub of dry ice, the freezer and on the counter top. No visible condensation of atmospheric water was observed on any of the samples cooled below 0 °C. The beans were equilibriated at each temperature for 2 hours prior to grinding.

The grinder was switched on 5 seconds before grinding and the beans were taken directly from their climates and fed into the hopper. The EK 43 is rated to grind 1200–1500 g/min, suggesting each 20 g dose of coffee was exposed to ambient conditions for no longer than 1 second. To prevent condensation of atmospheric water onto the surface of the *ground* coffee, the ground particulates were immediately placed into sample vials for laser diffraction particle size analysis. Absorption of atmospheric water proved not to be a problem, as duplicate samples which were exposed to atmospheric moisture as they equilibrated to room temperature, showed no difference to those that where sealed immediately upon grinding.

Each data set was obtained in triplicate, and each temperature was obtained in duplicate thereby generating 6 data sets per temperature. ANOVA was employed for determination of similarities in particle number distributions with consideration of the bean origin, processing method, roast and roaster included. The output of this statistical analysis is included in the supporting information.

## Do Differences in the Green Bean Affect the Final Grind?

The physical structure of roasted coffee beans is a complex composite of materials, containing high molecular weight fibrous molecules interspersed with amorphous and partially crystalline domains of a vast array of smaller organics. The extremely complex structure of both the roasted beans and grinding apparatus makes accurate first principles modeling a daunting prospect, and so fracturing is best studied experimentally (in line with previous studies of grinding other amorphous materials)^28^,^29^,^30^,^31^,^32^. That said, it could well be expected that the specific mix of chemicals that give different coffees their distinctive flavour may change the way in which the bean is fragmentised.

To investigate this, we elected to sample four speciality grade coffees. The selection spans the variables of origin, variety, processing method and roast profile, and is a representative cross section of contemporary speciality coffee. The four coffees described in Table 1 were ground at ambient conditions using the stipulated methods.

**Figure 3:**
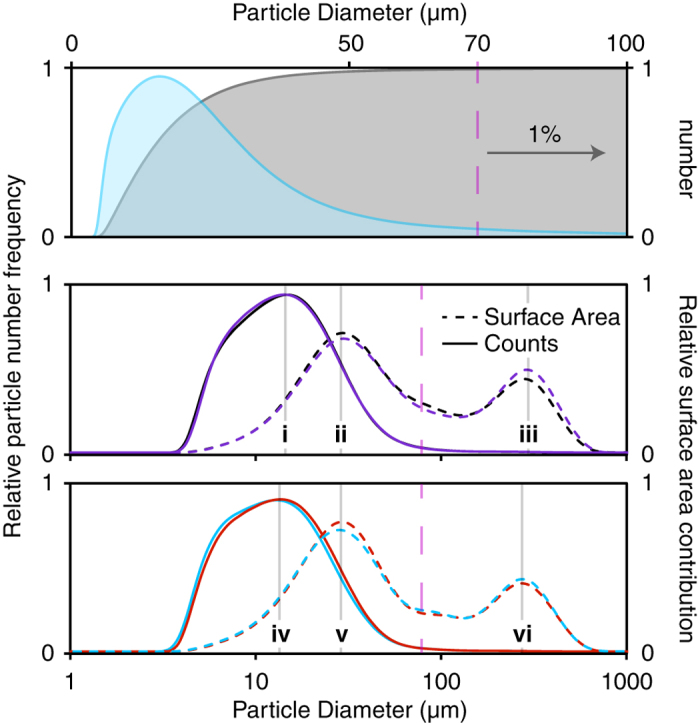
Upper panel: The particle size distribution as a function of number (cumulative) of physical particles (shown in blue) and the integral of this data (shown in grey), yields 99% of the particles with a diameter of 70 *μ*m or less. The fine particular cutoff is depicted as a purple dashed line. Middle and lower panel: The grind profiles of the four coffees examined here. The cumulative number and surface area contribution are shown in solid and dashed lines, respectively. The Tanzanian, Ethoipian, El Salvadorian and Guatemalan profiles are shown in black, purple, red and blue, respectively. Data modes **i**-**vi** are included for visual aid: **i** - 14.3, **ii** - 27.4, **iii** - 282.1, **iv** - 13.0, **v** - 27.4 and **vi** - 256.9 *μ*m.

Here we are concerned with the deviations in grind profile as a function of coffee origin, although before embarking on these experiments it was unclear what the grind profile looked like. The EK 43 produces particles ranging from 0.1 *μ*m to 1000 *μ*m, and whilst we have elected to present most of the data on a logarithmic scale, the linear scale is shown for the Tanzanian coffee in the upper panel of Fig. 3. All grind profiles appear as a skewed-Gaussian shape. In this case, we present the particle number distribution in the shaded blue region, and the integral in grey. We can arbitrarily define the fine particulate cutoff, graphically represented as a purple dashed line = *n* where:


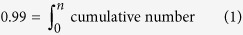


Here, *n* is a diameter in *μ*m. From the upper panel of Fig. 3, the Tanzanian *n* = 70 *μ*m (mode = 13.0 *μ*m, where the mode is the most frequent size occurrence). Given the skewed nature of the distribution, the mode is helpful in assigning key features of the distribution. However, it is not only the number of particles that contributes to the extraction of coffee, but also the available surface area obtained from these particles.

The grind profiles for the four coffees examined here are shown in the middle and lower panels of Fig. 3. They are presented on a logarithmic scale to accommodate the surface area contribution from the large particles. The surface area is estimated using a spherical approximation for the particles^33^, and is shown by the dotted line. Here, the data appears distinctly bimodal because the fine particulates contribute to the majority of the accessible surface area (modes **ii** and **v**), but large particulates (one/two orders of magnitude larger in diameter, **iii** and **vi**) are also present. These have an influence even at low concentrations.

There are minor differences in the grind profiles: The profiles shown in black and purple share similar particle number modes (**i**), and have a fine particulate cutoff of 76.4 ± 3.5 *μ*m. The profiles shown in red and blue produced a slightly finer particle distribution with a number mode (**iv**) 1.3 ± 0.7 *μ*m) more fine than the black/purple coffees, and a fine particulates cutoff of 69.6 ± 3.1 *μ*m. In summary, the coffees appear to produce a very similar grind distribution irrespective of the variables associated with bean production. Full ANOVA details are presented in Table S1. It should be noted that all of the beans considered here are roasted relatively ‘light’ compared to typical consumer grade coffee (although on the ‘Agtron Gourmet Scale’, these coffees all are catagorised as light-medium roast). We can only speculate how heavily decomposed beans (*e.g*. ‘dark’ or French roast) may deviate from these results; further experimentation is required to elucidate that effect.

For espresso, the coffee grinds can be thought of as a granular material, where the increase in pressure during tamping jams the materials^34^,^35^,^36^. The variability in particle size plays a significant role in the accessible surface area, but also in the vacuous space in which the water may flow through. From the work of Herman^37^, it is apparent that large particles install significant order of neighbouring small particles, which increases local density and therefore can result in inhomogeneous water flow through the espresso puck. However, given the subjectivity of coffee flavour and the preferences of practitioners working in the industry, it is not clear if there is an ideal particle size distribution: We only hope to shed light on the surprising consistencies between coffees.

Do Differences in the Roasted Bean Grind Temperature Affect the Final Grind? Temperature changes in amorphous materials can lead to well defined glass transitions, where the material changes from rubbery and flexible to being hard and brittle^38^. Some solids can also undergo shattering transitions, where there is an increased fragmentation rate as particle size decreases, resulting in production of greater numbers of fine particles^39^. This property is instigated by both temperature and crack velocity. It is understood that crystalline materials progress towards this shatter transition point with decreased temperature, because the strain on the lattice becomes proportionally larger with decreased lattice kinetics. However, roasted coffee is a complex material and glass or shattering transition points are unlikely to be constant across macroscopic regions of the bean, if present at all. Therefore, while it is reasonable to expect that a change in temperature will affect the grinding result, describing how and why this occurred is problematic. Experiment provides the simplest and most reliable route to assessing how temperature influences ground coffee particle size.

**Figure 4:**
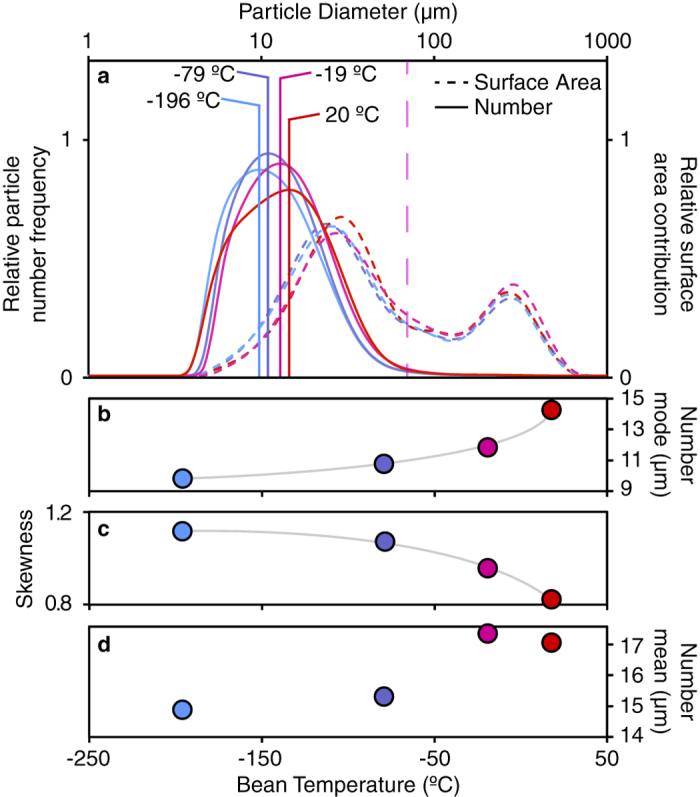
The temperature dependence on the grind profile of the El Salvadorian coffee, (**a**). The temperatures were achieved by grinding liquid nitrogen, dry ice, freezer and room temperature coffee, respectively. The fine particulate cutoff is schematically shown, with exact values corresponding to; −196 °C = 61 ± 3 *μ*m, −79 °C = 63 ± 3 *μ*m, −19 °C = 73 ± 3 *μ*m and 20 °C = 70 ± 3 *μ*m. The mode of the number distribution, (**b**) shows a clear and non-linear trend of increasing mode with increasing temperature. The distribution skewness is inversely proportional to temperature. From a flavour perspective this is a favourable feature because the surface area to volume ratio becomes increasingly significant for the smaller particles. The mean particle size, (**d**) is discontinuous with temperature likely indicating a transition between freezer and room temperature.

The lower the original bean temperature, the colder the produced particles will be at every stage of grinding. However colder bean fragments will absorb heat from their surroundings more quickly due to the larger temperature gradient, effectively reducing the indicated temperature difference between the samples. Therefore, the observed change in grind profile should be considered a lower limit on the effects of grinding at reduced temperatures. Given the inhomogeneous nature of the beans, it is likely that cooling the burrs (and hence further reducing the temperature of the particles as they are ground) would smoothly continue the trend observed in Fig. 4.

Some fraction of particles are produced in their final size from the initial fracturing of the whole bean (or large portion thereof), and so are truly produced at the stated temperature. However, experiments using a single impact event (*i.e*. hitting a cold bean with a mallet), show that only a small amount of small particles are produced on initial bean fracturing, so most particles do have some time for thermalisation before further fracturing occurs.

Even with some particle thermalisation due to room temperature burrs, the initial bean temperature has a significant effect on the modal particle size distribution (Fig. 4a) reducing the mode by 31% as the beans are cooled from room temperature to −200 °C, as shown in Fig. 4b. Additionally, the distribution generally becomes narrower as the beans are cooled (Fig. 4c) with the biggest change occurring between room temperature and −19 °C beans. The room temperature grind profile is also distinctly less Gaussian-like, with the development of a hip at approximately 9.5 *μ*m. This detail could indicate that some components of the bean undergo a shattering transition between 20 °C and −19 °C, and studies are ongoing into the origin of this feature.

To probe the reversibility of this transition, we performed the same room temperature experiments with coffee beans that had been cooled to liquid nitrogen temperatures and then allowed to reheat to room temperature. It appears that if there is a transition, it is reversible as there were no notable differences between the two samples. This is not surprising given the very low water concentration in roasted coffee: The thermal contraction and re-expansion of coffee did not play a significant role in the grind profile obtained from either test set.

## Applications and Concluding Remarks

In busy coffee shops, it is common practice to reduce burr grinding aperture as the day progresses in order to produce a consistent cup of coffee. From work presented here, we propose that this phenomenon is a direct product of the grinding burrs (and potentially beans sitting in the hopper in grinders other than the EK 43) becoming increasingly warm as the grinder is used. The particle warming at the interface between the coffee bean and warm burr - which can certainly be much higher in temperature than explored in this study - shifts both the mode and spread of the particle size distribution. Thus, as the grinder gets warm a finer grind setting may be required to obtain the same effective surface area as the same coffee ground on cooler burrs. However, we also observe a difference in the shape of the distribution with temperature, which indicates that simply grinding finer with warm burrs will not produce the same result as grinding coarsely with cold burrs. The impact on taste and preference is not the focus of this study, but is certainly an interesting avenue to explore in the future.

The distinct lack of dependence on origin and processing method is comforting for coffee shops that serve coffees from multiple origins, and also for roasters who develop and market blends (mixtures of origins). One grand challenge with blended coffee is to produce a product where each desired component is equally soluble, such that the cup of coffee tastes appropriately extracted. Consider the traditional blend of Brazilian and Ethiopian coffees: The two are combined to obtain the body and nuttiness from the Brazilian, and the fruit and complexity from the Ethiopian. But such results are only obtained if both beans reach terminal extraction at similar rates. Here, we have minimised one variable by showing that at least the accessible surface area is kept constant whilst grinding, thereby placing the chemical problems associated with blending solely on the roast profile.

From a physical chemistry perspective, the temperature dependence presents many interesting questions. Given the minimal difference between liquid nitrogen and dry ice temperatures and the reversibility of the cooling, we question whether it is possible in the future to cryogenically store roasted coffee at these temperatures. Indeed, water content in the roasted bean is of paramount importance at these temperatures, as water expansion may lead to be fracturing. Also, prolonged exposure to water can result in the solvation of flavoursome molecules, thereby decreasing the lifetime of the frozen product. But if these variables were managed, there are a host of subsequent implications for the storage and relative quality assessment allowing for access to direct year-to-year comparison of crop quality. From a consumption perspective, cooling of coffee beans significantly decreases the rate of mass loss through volatile sublimation/evaporation. Thus, coffee that is ground and brewed cold could potentially demonstrate increased aroma and or flavour in the eventual brewed cup.

From an industrial perspective, the yield of extraction is paramount. Grinding colder coffee beans produces a more uniform particle distribution, with a decreased particle size. While the decreased particle size will tend to speed up extraction due to the larger surface area, the increased uniformity should minimise the amount of wasted bean, which is discarded without being extracted to completion. Whilst active cooling of either the coffee beans or burrs is energy consuming, the benefit of cold coffee grinding may offset this cost with more efficient extraction from the smaller particles.

## Additional Information

**How to cite this article**: Uman, E. *et al*. The effect of bean origin and temperature on grinding roasted coffee. *Sci. Rep*. **6**, 24483; doi: 10.1038/srep24483 (2016).

## Supplementary Material

Supplementary Information

## Figures and Tables

**Figure 1:**
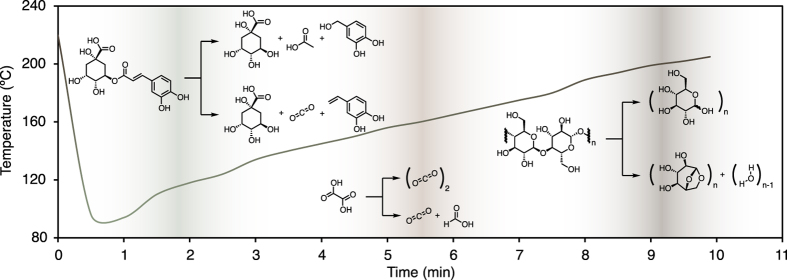
The roast profile for the Tanzanian Burka (Has Bean). In this case, 10 kg of the Burka coffee was roasted in a 12 kg Probat Roaster. The temperature was monitored with a probe in the headspace of the oven, and hence the hot air rapidly cools due to thermal energy transfer to the green coffee. The temperature trajectory throughout the roasting process determines the decomposition of organic materials in coffee. Three illustrative decomposition reactions are shown that are representative processes throughout the heating process. At lower temperature a chlorogenic acid (left) may decompose through either hydrolysis or pyrolysis into quinic acid, acetic acid and the phenolic compound 3,4-dihydroxybenzyl alcohol^40^, or quinic acid, carbon dioxide and 3,4-dihydroxystyrene^41^,^42^. Oxalic acid (centre) may decarboxylate to either CO_2_ or in the case of incomplete combustion CO_2_ and formic acid^19^. At higher temperatures cellulose can undergo hydrolysis to smaller sugar derivatives including glucose and levoclucosan^43^,^44^,^45^. Both the temperature and time determine the chemical composition of the roasted coffee: In this case, the coffee was removed from the oven after 9 m 54 s as this time was determined to result in a soluble, sweet and favourably acidic product.

**Table 1:** Details on the four coffees (Coffea arabica) that were ground in this experiment: two African and two South American.

Farm/Estate	Origin	Variety	Roaster	Roast	Agtron colour
Las Ilusiones (W)	Guatemala	Caturra and Bourbon	Round Hill	Espresso	62
Santa Petrona (W)	El Salvador	Pacamara	Has Bean	Espresso	59
Burka (W)	Tanzania	Red Bourbon	Has Bean	Espresso	59
Sasaba (N)	Ethiopia	Mixed Heirloom	James Gourmet	Filter	68

(W) and (N) indicate washed and natural processing methods, respectively. ‘Roast’ is used as an indication for whether the coffee was roasted for filter (lighter) or espresso (darker) style coffee, and can be quantified by the ‘Agtron colour’ as determined by the Agtron spectrophotometeric measurement^46^. All coffees examined here would be considered light/medium roasted relative to typical commodity grade coffee.
