# Supplementary material for: The effect of bean origin and temperature on grinding roasted coffee
Source: Sci Rep. 2016 Apr 18;6:24483. doi: 10.1038/srep24483 (PMC4834475; doi:10.1038/srep24483)
Supplement: Supplementary Information [file srep24483-s1.doc]

Supporting Information:

The effect of bean origin and temperature on grinding roasted coffee

Erol Uman ^a^, Maxwell Colonna-Dashwood ^b^, Lesley Colonna-Dashwood ^b^, Matthew Perger ^c^, Christian Klatt ^d^, Stephen Leighton ^e^, Brian Miller ^a^, Keith T. Butler ^f^, Brent C. Melot ^g^, Rory W. Speirs ^h^, Christopher H. Hendon ^i^*

*^a^ Meritics Ltd., 1 Kensworth Gate, Dunstable, LU6 3HS, United Kingdom*

*^b^ Colonna and Smalls, 6 Chapel Row, Bath, BA1 1HN, United Kingdom*

*^c^ St Ali / Sensory Lab, 12-18 Yarra Pl, South Melbourne, Victoria, 3205, Australia*

*^d^ Mahlkönig GmbH & Co.KG, Tilsiter Str. 142, 22047 Hamburg, Germany*

*^e^ Has Bean Coffee Ltd., Unit 16, Ladford Covert, Stafford, ST18 9QL, United Kingdom*

*^f^ Department of Chemistry, University of Bath, BA2 7AY, Bath, United Kingdom*

*^g^ Department of Chemistry, University of Southern California, Los Angeles, CA 90089, United States of America*

*^h^ School of Physics, The University of Melbourne, Victoria, 3010, Australia*

*^i^ Department of Chemistry, Massachusetts Institute of Technology, Cambridge, MA 02139, United States of America*

* hendon@mit.edu

**Table S1** One way ANOVA for the comparison of bean origin and processing particle count grind size distributions.

| **Analysis of Variance (One-Way)** | | | | | | |
| --- | --- | --- | --- | --- | --- | --- |
|  |  |  |  |  |  |  |
| **Summary** |  |  |  |  |  |  |
| *Groups* | *Sample size* | *Sum* | *Mean* | *Variance* |  |  |
| *Burka* | 94 | 99.99345 | 1.06376 | 3.41717 |  |  |
| *Las Il* | 94 | 99.99755 | 1.0638 | 3.3637 |  |  |
| *Santa* | 94 | 99.99775 | 1.06381 | 3.34866 |  |  |
| *Sasaba* | 94 | 99.99506 | 1.06378 | 3.45942 |  |  |
|  |  |  |  |  |  |  |
| **ANOVA** |  |  |  |  |  |  |
| *Source of Variation* | *SS* | *df* | *MS* | *F* | *p-level* | *F crit* |
| Between Groups | 1.36879E-7 | 3 | 4.56262E-8 | 1.34304E-8 | 1. | 2.6289 |
| Within Groups | 1,263.77209 | 372 | 3.39724 |  |  |  |
|  |  |  |  |  |  |  |
| *Total* | 1,263.77209 | 375 |  |  |  |  |


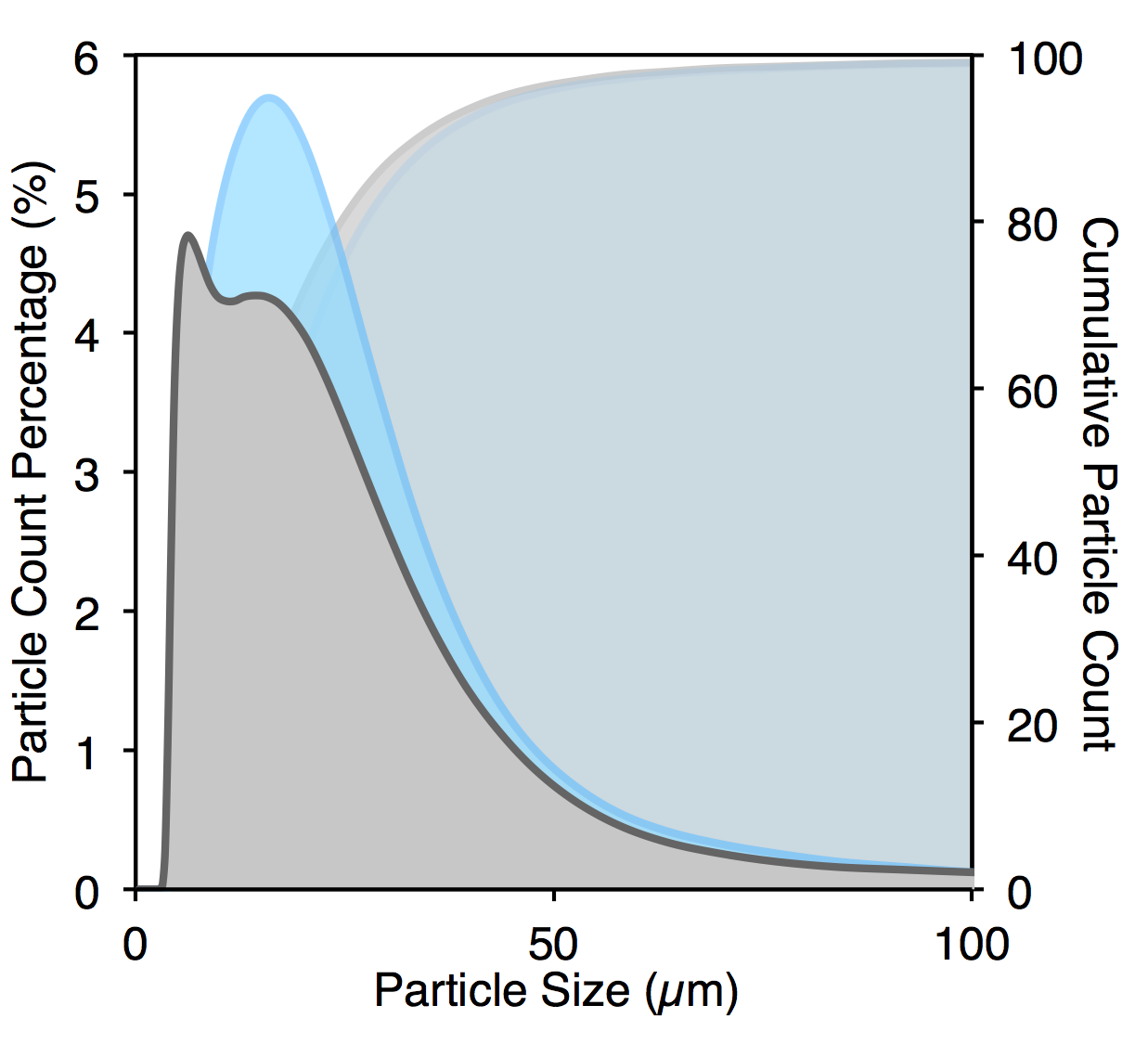


Figure S1 A comparison grind size distributions of two EK 43 grinders fitted with Turkish burrs. Whilst the grinders were not set to grind at the same aperture due to the arbitrary alignment of the external dial, the bimodal grey distribution is indicative of misaligned burrs. We elected to use the grinder that produced the blue curve in these studies.
